# Impact of an Electronic Medical Record–Connected Questionnaire on Efficient Nursing Documentation: Usability and Efficacy Study

**DOI:** 10.2196/51303

**Published:** 2023-09-25

**Authors:** Kana Kodama, Shozo Konishi, Shirou Manabe, Katsuki Okada, Junji Yamaguchi, Shoya Wada, Kento Sugimoto, Sakiko Itoh, Daiyo Takahashi, Ryo Kawasaki, Yasushi Matsumura, Toshihiro Takeda

**Affiliations:** 1 Department of Medical Informatics Osaka University Graduate School of Medicine Suita Japan; 2 Department of Transformative System for Medical Information Osaka University Graduate School of Medicine Suita Japan; 3 MKS Inc Osaka Japan; 4 Department of Home Health and Palliative Care Nursing Graduate School of Health Care Sciences Tokyo Medical and Dental University Tokyo Japan; 5 Division of Public Health Department of Social Medicine Osaka University Graduate School of Medicine Suita Japan; 6 National Hospital Organization Osaka National Hospital Osaka Japan

**Keywords:** nursing system, electronic questionnaire, electronic medical record, medical informatics, EMR, medical records, EHR, health record, health records, nursing, documentation, documenting, usability, self-reported, patient data, questionnaires, data conversion, nursing record, nursing records, data capture, information system, information systems

## Abstract

**Background:**

Documentation tasks comprise a large percentage of nurses’ workloads. Nursing records were partially based on a report from the patient. However, it is not a verbatim transcription of the patient's complaints but a type of medical record. Therefore, to reduce the time spent on nursing documentation, it is necessary to assist in the appropriate conversion or citation of patient reports to professional records. However, few studies have been conducted on systems for capturing patient reports in electronic medical records. In addition, there have been no reports on whether such a system reduces the time spent on nursing documentation.

**Objective:**

This study aims to develop a patient self-reporting system that appropriately converts data to nursing records and evaluate its effect on reducing the documenting burden for nurses.

**Methods:**

An electronic medical record–connected questionnaire and a preadmission nursing questionnaire were administered. The questionnaire responses entered by the patients were quoted in the patient profile for inpatient assessment in the nursing system. To clarify its efficacy, this study examined whether the use of the electronic questionnaire system saved the nurses’ time entering the patient profile admitted between August and December 2022. It also surveyed the usability of the electronic questionnaire between April and December 2022.

**Results:**

A total of 3111 (78%) patients reported that they answered the electronic medical questionnaire by themselves. Of them, 2715 (88%) felt it was easy to use and 2604 (85%) were willing to use it again. The electronic questionnaire was used in 1326 of 2425 admission cases (use group). The input time for the patient profile was significantly shorter in the use group than in the no-use group (*P*<.001). Stratified analyses showed that in the internal medicine wards and in patients with dependent activities of daily living, nurses took 13%-18% (1.3 to 2 minutes) less time to enter patient profiles within the use group (both *P*<.001), even though there was no difference in the amount of information. By contrast, in the surgical wards and in the patients with independent activities of daily living, there was no difference in the time to entry (*P*=.50 and *P*=.20, respectively), but there was a greater amount of information in the use group.

**Conclusions:**

The study developed and implemented a system in which self-reported patient data were captured in the hospital information network and quoted in the nursing system. This system contributes to improving the efficiency of nurses’ task recordings.

## Introduction

Nurses perform various tasks such as direct care of patients, patient-family relations, documentation, preparation of medications, and meetings, and documentation of electronic health records (EHRs) accounts for as much as 13%-25% of their total work time [1-3]. In general, nurses are required to create records and documents for each patient on the day of admission. Estimations of the contribution to a reduction in documentation time by EHR or computational order vary by study [4,5]. In recent years, there have been some reports on initiatives using new technologies, such as voice recognition; however, they are still in the prepractical stage or have only been demonstrated on a small scale [6,7]. In addition, all these reports are of devices on the provider's side and do not involve patients to reduce the recording time for nurses.

Nursing records contain extensive patient information. When patients are scheduled for inpatient care, they are asked to complete a paper questionnaire on a wide range of activities of daily living (ADL) and habitats. The nurse in charge, sometimes supplementing the patient's responses by interviewing in person, performs a comprehensive nursing assessment of the inpatient, which consists of ADL, instrumental activities of daily living, use of assistive devices and braces, dietary restrictions, appetite, weight change, bedsores, skin disorders, drinking habits, and smoking habits. These assessments are registered in the nursing system as part of the patient profile.

Any report on the status of a patient’s health condition that comes directly from the patient without interpretation of the patient’s response by a clinician or anyone else is called a patient-reported outcome (PRO) [8]. Questionnaires answered by the patients were also considered a type of PROs. In recent years, PROs have been increasingly collected using electronic devices and are now called as electronic PROs [9]. The integration of PROs into EHRs is difficult [10]; thus, there have been few attempts to integrate PROs into EHR [11-13]. In particular, in Japan, the situation is more difficult because electronic medical records (EMRs) in hospitals are usually on premise and external connectivity is considerably limited. Furthermore, extant reports have not clarified whether the integration can improve the workflow of health care providers. First, although they would be based on patient reports, nursing records are a type of clinical professional record. They are not a verbatim transcription of the patient's complaints. In other words, it is not sufficient to import PROs into the EHR. Appropriate mapping and conversion are required.

In this context, this study developed a novel system in which patient responses to electronic questionnaires are securely captured in the EHR and quoted in the forms of the nursing system in the EMR; it examined whether this system would reduce the time nurses spend completing patient profiles in the nursing system.

## Methods

### Overview

**Figure 1:**
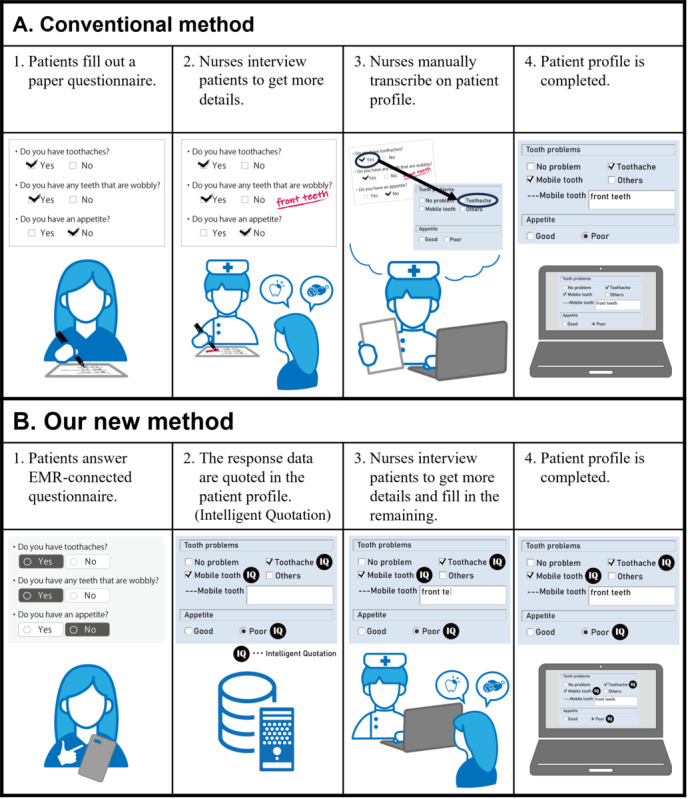
Overview. (A) Conventionally, the patient profile is typically completed as follows: (1) patients fill out a paper questionnaire, (2) nurses interview a patient for more information, (3) nurses manually transcribe the patient profile, and (4) the patient profile is completed. (B) In our new method, the patient profile is typically completed as follows: (1) patients answered an EMR-connected questionnaire, (2) the response data are mapped to the corresponding forms of the patient profile in a predefined manner, which is called Intelligent Quotation, (3) nurses interview the patient for more information and fill in the remaining, and (4) the patient profile is completed. The “IQ” icon indicates the items for which data are quoted from the EMR-connected questionnaire. EMR: electronic medical record.

This study aimed to enable nurses to complete patient profiles with minimal recording work. An overview of this process is shown in Figure 1. The patient profile is one of the nursing records; it is a comprehensive nursing assessment of an inpatient consisting of 77 forms. Conventionally, patients are asked to complete paper-based questionnaires. Subsequently, the nurses interviewed the patients for more information. Finally, the nurse transcribed all data into a patient profile. Using our new method, the patient first responds to an EMR-connected questionnaire. The patient response data are stored on the EMR server and quoted in the corresponding forms of the patient profile in a predefined manner (Intelligent Quotation). Thus, nurses do not need to transcribe patient response data manually. Consequently, the nurse can complete the patient profile by interviewing the patient and completing the remaining parts of the form. The system was implemented using a preadmission nursing questionnaire. The details of this process are described below.

### EMR-Connected Questionnaire System

**Figure 2:**
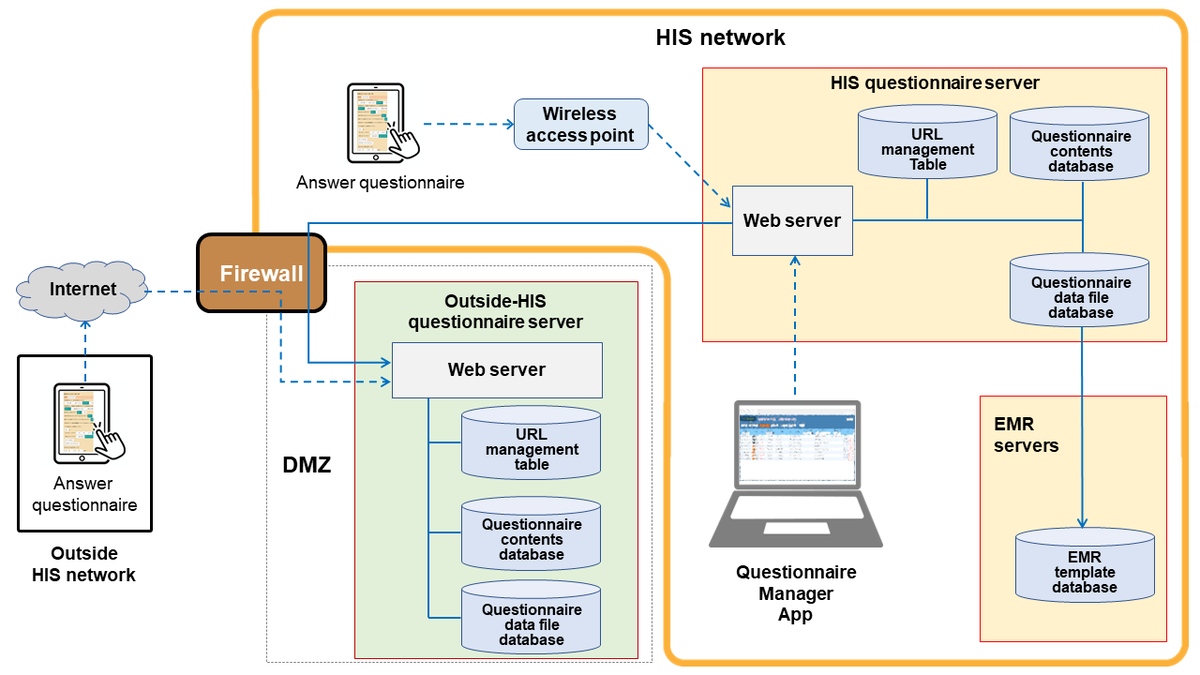
Overall view of EMR-connected questionnaire system. Patients answer electronic questionnaires inside and outside the HIS network, and the data are securely stored within the HIS network and in the EMR servers. EMR: electronic medical record; HIS: hospital information system.

A web app, EMR-connected questionnaire, was developed and implemented (Figure 2), and patients can answer the questionnaire anywhere using tablets or smartphones. Generally, patients respond to a questionnaire using a hospital tablet connected to the hospital information system (HIS) network. The patient response data were directly transmitted to the HIS questionnaire server in the HIS network. Outside the HIS network, patients can answer the questionnaire via the internet using their smartphones or tablets (Outside-HIS use).

The questionnaire contents were registered in advance using the HIS Questionnaire Server. A unique identifier and URL are generated each time a medical provider specifies the target patient and questionnaire to be used in the Questionnaire Manager App. They did not contain patients’ identifying information. They were registered in the URL Management Table in the HIS Questionnaire Server. A QR code with an embedded URL was issued, and when it was scanned by a hospital tablet, the questionnaire content was displayed. The patients answered the questionnaire, and the response data were sent directly to the HIS Questionnaire Server. After the medical provider checks for completeness using the Questionnaire Manager App, the data are registered in the EMR Template Database.

The data flow of Outside-HIS use differs partially from that of HIS Use in ensuring a secure transmission function to acquire patient questionnaire responses. The questionnaire content was registered in advance using the HIS/Outside-HIS Questionnaire Server. As with HIS Use, an identifier and URL are generated each time a provider specifies the target patient and questionnaire to be used in the Questionnaire Manager App. The identifier and URL were written to the URL Management Table on the HIS and the Outside-HIS Questionnaire Servers. However, for privacy protection, patient information was not included in the URL Management Table on the Outside-HIS Questionnaire Server. A QR code with an embedded URL was issued and printed onto the patient. When a patient scans a QR code with their smartphone at home or elsewhere, the content is displayed. The patients answered the questionnaire, and their response data were transmitted through the firewall to the Outside-HIS Questionnaire Server. For security reasons, firewalls do not accept external connections. Response data from the Outside-HIS Questionnaire Server were transmitted into the HIS Questionnaire Server. Subsequently, the data file was removed from the Outside-HIS Questionnaire Server. Patient information was assigned by referring to a URL Management Table. The subsequent process was the same as that for HIS Use.

Through these processes, the patients’ questionnaire response data were stored as XML in the EMR Template Database. The stored data can be further quoted for applications such as nursing systems and progress notes in the EMR [14].

### Preadmission Nursing Questionnaire

The contents of the electronic medical questionnaire were created arbitrarily according to the intended use. The study had already implemented a system for the preadmission nursing questionnaire. One of the researchers of this study, a registered nurse, selected items from the patient profile, that were easy for patients to answer using electronic devices, converted them into patient-friendly wording, and drafted the questionnaire. Further discussions with the nursing staff led to brushing up on the preadmission nursing questionnaire. It consists of 15 questions regarding ADLs (seeing, hearing, walking, changing clothes, bathing, toileting, etc), weight change, dietary restrictions, drinking habits, and smoking habits. As the items in the questionnaire change depending on the answers, the patient may be asked to respond to another layer of detailed questions. Patients answered a minimum of 15 items and a maximum of 53. Most questions were single- or multiple choice questions. Some items, such as the amount of weight change, were answered by entering numbers on the screen using the keypad. The patients could answer the questionnaire in the hospital or at home as per their preference.

### Intelligent Quotation of Questionnaire Response Data in Patient Profiles

Hospital nurses performed a comprehensive nursing assessment of the inpatients based on Gordon's Functional Health Patterns [15]. The assessment is registered in the nursing system as a patient profile using single choice, multiple choice, or text-entry forms. The patient profile comprised 77 sections including health perception/health management, nutrition, elimination, physical activity and exercise, sleep and rest, cognition and perception, self-perception and self-concept, role and relationship, sexuality and reproduction, coping or stress tolerance, and values and beliefs (see Table S1 in Multimedia Appendix 1). Assessment items in each section were added depending on the patient.

As mentioned above, when a patient answers an electronic questionnaire, the data are stored in an EMR database. When the nurse creates a patient profile, the application is launched with the patient's response data already mapped to some of the corresponding templates of the forms that create the patient profile. In the mapping, the following well-designed methods were used to ensure that the patient self-reports were accurately reflected in the nursing records. Thus, nurses could fill in the remaining portions and, if necessary, correct the mapped data. In 20 of the 77 sections, the patient response data were quoted to a greater or lesser extent using the following techniques: from the perspective of Gordon’s functional health patterns, quotes were set up in 5 of the 11 patterns: health perception or management, elimination, physical activities or exercise, nutrition or metabolism, and cognition or perception. No quotations were provided for the remaining 6 patterns: sleep and rest, self-perception and self-concept, role and relationships, sexuality and reproduction, coping or stress tolerance, or values and beliefs.

### Converting to Medical Terminology and Aggregation of Information

**Figure 3:**
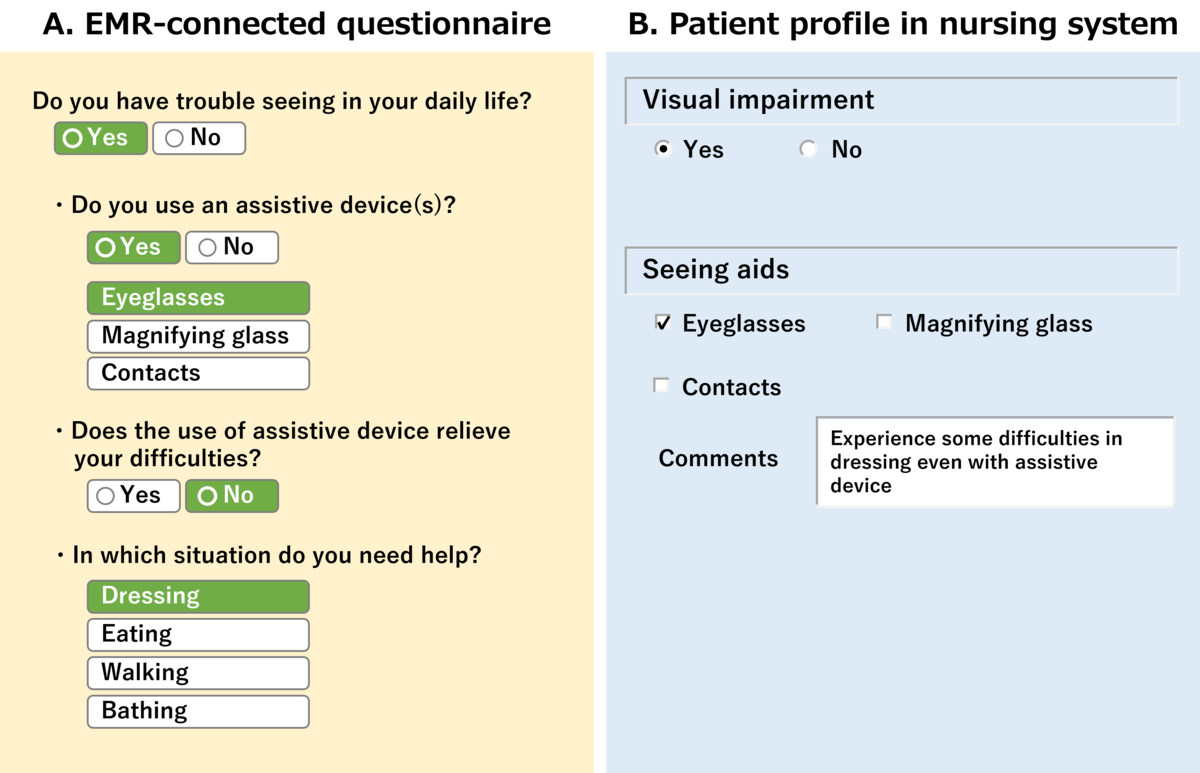
Gathering information. (A) In the EMR-connected Questionnaire, a patient answers the questions sequentially. (B) The response data are mapped to the corresponding items in the patient profile of the nursing system. Some responses are combined into one. “Comment” is also generated by integrating the answers to the two questions, whether and when they have difficulties even with assistive devices. EMR: electronic medical record.

The electronic questionnaire was patient oriented and contained redundant patient expressions. These were appropriately mapped to the corresponding medical terms in the nursing system (Textbox 1). The nursing system also gathered information by integrating the responses to several questions in the questionnaire (Figure 3).

**Expression in the electronic medical record-connected questionnaire and corresponding terminology in the patient profile on the nursing system. Redundant expressions for patients in the electronic medical record-connected questionnaire are converted to appropriate terminology in the patient profile on the nursing system.**

**Electronic medical record–connected questionnaire**

- I can do it myself
- I can do it with someone’s assistance
- I cannot do it myself
- Do you have trouble hearing in your daily life?
- Have leg weakness
- Told by a doctor not to put weight on either leg
- Spend most of my time on the bed

**Patient profile in the nursing system**

- Independent
- Needs help
- Unable
- Hearing impairment
- Lower extremity weakness
- Weight-bearing restriction
- Activity intolerance

### Calculation

**Figure 4:**
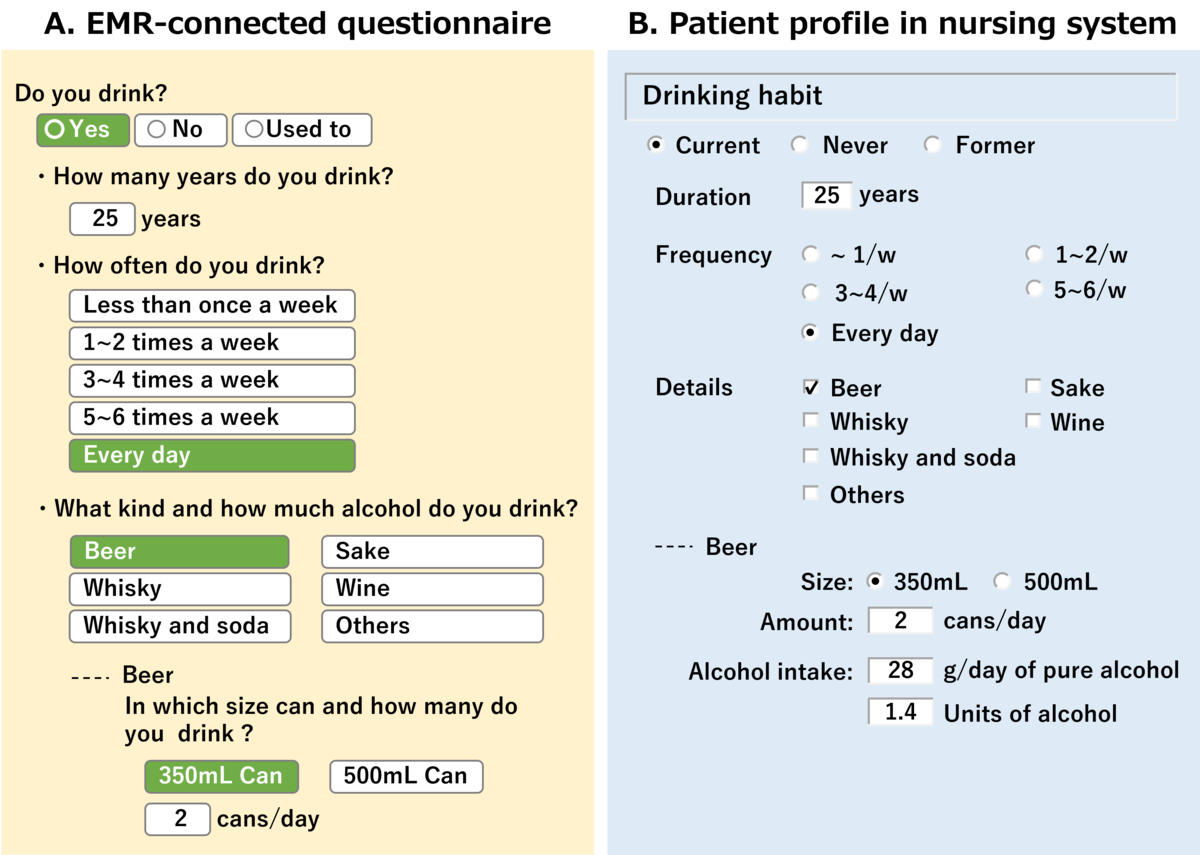
Calculation. (A) In the EMR-connected questionnaire, a patient answers the details about their drinking habits. (B) In the patient profile, the amount and units of alcohol intake are automatically calculated based on the patient’s answer. For beer, pure alcohol is calculated by the equation: volume of can×number of cans×0.8×0.05. One unit of alcohol is equal to 20 g of pure alcohol. EMR: electronic medical record.

Patient responses to electronic questionnaires can be used to calculate the clinically relevant indicators in the nursing system. For example, smoking duration can be calculated from patients’ answers regarding the age at which they started smoking and the age at which they quit smoking. The amount of alcohol intake per day and alcohol units (20 g of alcohol is defined as 1 unit in Japan) can also be calculated from the patients’ answers regarding their drinking habits (Figure 4).

### Combination of Quotation and Direct Interview

**Figure 5:**
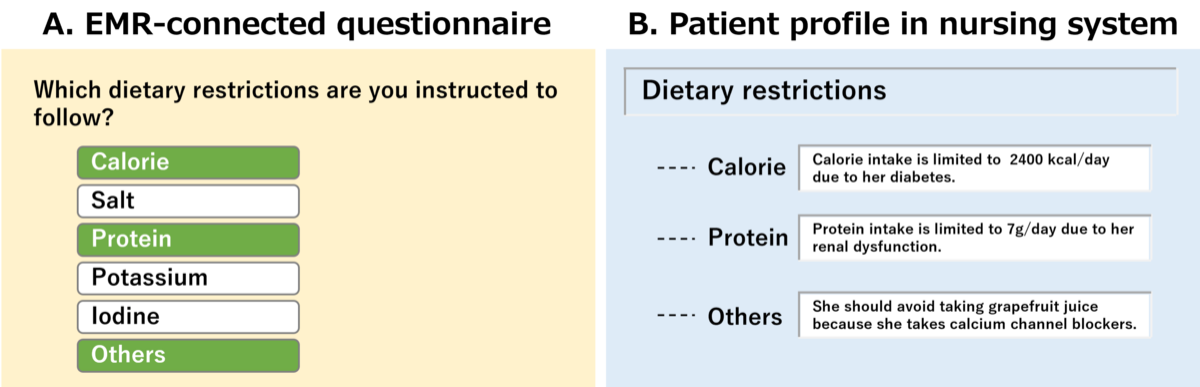
Combination of quotation and direct interview. (A) In the EMR-connected questionnaire, a patient answers what dietary restriction they have. In this example, calorie, protein, and others are chosen. (B) In the patient profile, a textbox for manual typing appears according to the patient’s response. In this example, text boxes for calories, protein, and others appeared. Details are filled in the boxes after nurses conduct a focused interview with the patient. EMR: electronic medical record.

Given that electronic questionnaires were answered using tablets or smartphones, they were not suitable for patients to answer in detail using text input. For some assessment items, the patients first answered the electronic questionnaire's multiple choice questions, and the responses were quoted in the nursing system. However, further details are lacking in this regard. Therefore, the nurse conducted a focused interview to obtain details that should be registered in the patient profile (Figure 5). Thus, the patient profile was efficiently completed by combining the quotations of the questionnaire response data and the nurses’ interviews.

### Usability Assessment

Usability was measured according to criteria defined by the ISO (International Organization for Standardization) 9241-11 standard [16]. A survey was administered to patients who completed an electronic medical questionnaire. The effectiveness was evaluated based on whether the patients answered the medical questions. Regarding satisfaction, the patients rated the ease of entry and their willingness to use the system again on a 5-point scale. Patient attributes such as age and sex were extracted from the EMR.

### Contribution to Reduction of Input Time for Patient Profiles

The time nurses spent on the day of admission, as well as the following day entering the comprehensive inpatient assessment into the nursing system (input time), was calculated using the operation logs of the EMR system for cases with scheduled admissions from August to December 2022. Patients previously admitted to the hospital were excluded from the study. The total time was calculated when the nurse created the patient profile in several small steps. Nurses’ EMR tasks are sometimes interrupted by other priority events, leading to task switching [17]. If a nurse was in the middle of entering the patient profile and left without saving it or logging out, the input time may have been overestimated. Therefore, input times 3 times greater than the mean by SD were considered outliers and excluded from the analysis. However, the study also conducted an analysis that did not exclude outliers and discussed them in the supplementary text. Patient attributes such as age and sex were extracted from the EMR.

Patients were divided into 2 groups according to whether they had completed a preadmission electronic questionnaire. Between the 2 groups, the number of single and multiple-choice questions and characteristics entered into the comprehensive inpatient assessment and the patient’s profile, such as age, sex, cognitive impairment, and admission ward category, were analyzed. Cognitive impairment was collected from diagnosis procedure combination data [18]. Patient ADL on admission and prolonged hospitalization were also included in the analysis as indicators of case complexity. The bedriddenness rank is a reliable ADL scale scored by medical professionals [19]. It consists of 4 major categories: J, A, B, and C (J being independent and A, B, and C indicating less independence in that order). In this study, category J was defined as independent ADL and the others were dependent ADL. A prolonged hospital stay was defined as a case in which its hospital stay exceeded period 2, that is, the average hospital stay under the diagnosis procedure combination code [18]. Moreover, “years working in nursing” and “EMR use duration > 3 years” were also analyzed.

### Ethical Considerations

This study conformed to the ethical guidelines outlined in the Declaration of Helsinki. The study protocol was approved by the Ethical Review Board at Osaka University Hospital (20247). All patients in the effectiveness and satisfaction assessments were given an adequate explanation and provided their informed consent electronically. In the efficacy assessment, individual consent was waived with the permission of the ethics committee; however, participants were given the right to veto consent by opting out.

### Statistical Analysis

R (version 4.2.2; R Foundation) was used for the statistical analysis. Continuous variables were expressed as medians and quartiles, and categorical variables were expressed as frequencies and proportions (%). The Wilcoxon rank-sum test was used to test continuous variables, and the Pearson chi-square test was used to test categorical variables. The Cochran-Armitage test was used to test the trends of the proportions.

## Results

### Usability Assessment

Responses were obtained from 4083 patients scheduled for hospitalization between April and December 2022 who agreed to participate in a usability survey. They had a median age of 60 years; 2073 (51%) were men, and 3613 (92%) were daily users of smartphones, tablets, or personal computers. Of all patients, 3111 (78%) completed the electronic medical questionnaire by themselves. Factors associated with patients requiring assistance completing the questionnaire were not being comfortable using digital devices (*P*<.001) and patient's age (responses by proxy aged <20 years and in their 20s, 30s, 40s, 50s, 60s, 70s, and ≥80 years were n=407, 80%; n=18, 10%; n=9, 4%; n=15, 3%; n=27, 4%; n=55, 8%; n=187, 20%; and n=183, 49%, respectively; *P*<.001). When patients did not respond, their parents responded on their behalf for patients younger than 20 years and their children for those older than 70 years. Proxy respondents consisted of parents (n=398, 98%) for patients younger than 20 years and children (n=231, 62%) and spouses (n=79, 21%) for those older than 70 years. Of the patients who answered the electronic questionnaire themselves, 2715 (88%) gave a score of 4 or 5 for ease of entry, and 2604 (85%) gave a score of 4 or 5 for their willingness to use it in the future (Table 1). Factors associated with patients who reported an ease of entry score of 3 or less were being an older adult (*P*<.001) and not being habituated to using digital devices (*P*<.001).

**Table 1:** Surveys on ease of entry and willingness to use again for patients who answered the questionnaire themselves^a^.

Score	Ease of entry, n (%)	Willing to use it again, n (%)
5	2020 (66)	1982 (65)
4	695 (23)	622 (20)
3	304 (9.9)	348 (11)
2	21 (1)	60 (2)
1	32 (1)	31 (1)

^a^5 is the highest score and 1 is the lowest score.

### Contribution to the Reduction of Input Time for Patient Profiles

Of the 2425 cases, 57 were excluded from the analysis because the input time was an outlier. Of the remaining 2368 cases, 1287 were in the use group and 1081 were in the no-use group; the use group had a shorter input time, more multiple choice questions, and more text-entry questions with more characters entered than the no-use group (Table 2). There was no difference in age, sex, prolonged hospital stay, or cognitive impairment between the 2 groups, but there was a difference in the admission ward and patient ADL. Subsequently, a stratified analysis was performed according to the admission ward and patient ADL. In the analysis by admission wards (Table 3), there was no difference in the number of questions or characters in the patient profile between the 2 groups in the internal medicine ward. Nonetheless, the use group took less time (2 minutes, 18%) to complete the profile than the no-use group. By contrast, in the surgical wards, there was no difference in the input time between the 2 groups, but the number of questions and characters in the patient profiles created by the use group was greater than those in the no-use group. In the analysis by patient ADL (Table 4), there was no difference in the input time in the patient with independent ADL, but the number of questions and the number of characters in the patient profiles created by the use group was greater. Conversely, in the patient with dependent ADL, the use group took comparatively less time (1.3 minutes, 13%) to complete the patient profile. An analysis using all measurements without excluding outliers in the input time was also performed, and the results were similar (Tables S2-S4 in Multimedia Appendix 1).

**Table 2:** Time required for nurses to enter patient data in the nursing system.

Characteristic			Use group (n=1287)	No-use group (n=1081)		*P* value	
Patient age (year), median (IQR)			58 (41-73)	56 (33-73)		.14	
Patient sex (male), n (%)			592 (46)	489 (45)		.71	
Independent ADL^a^, n (%)			885 (84)	703 (74)		<.001	
Prolonged hospital stay, n (%)			666 (54)	527 (52)		.37	
Cognitive impairment, n (%)			89 (6.9)	82 (7.6)		.54	
Single-choice items registered, median (IQR)			56 (50-63)	56 (50-63)		.25	
Multiple choice items registered, median (IQR)			16 (12-20)	15 (11-19)		.03	
Sum of characters entered, median (IQR)			237 (152-353)	208 (139-299)		<.001	
Input time (minutes), median (IQR)			9.4 (6.2-15.0)	10.1 (6.6-16.7)		.007	
**Ward, n (%)**							<.001
	Internal medicine	593 (46)		266 (25)	N/A^b^		
	Surgery	694 (54)		815 (75)	N/A		
Years working in nursing, median (IQR)			4.7 (2.4-12.4)	4.6 (2.2-10.6)		.26	
EMR^c^ use duration > 3 years, n (%)			834 (65)	690 (64)		.62	

^a^ADL: activities of daily living.

^b^N/A: not available.

^c^EMR: electronic medical record.

**Table 3:** Time required for nurses to enter patient data in the nursing system stratified by the patient wards.

Characteristic			Use group		No-use group		*P* value
**Internal medicine, median (IQR)**							
	Single-choice items registered	55 (49-63)		54 (49-63)		.78	
	Multiple choice items registered	15 (12-19)		15 (11-19)		.49	
	Sum of characters entered	222 (144-331)		216 (122-332)		.27	
	Input time (minutes)	8.9 (6.0-14.6)		10.9 (7.1-19.0)		<.001	
**Surgery, median (IQR)**							
	Single-choice items registered	56 (50-64)		57 (51-64)		.47	
	Multiple choice items registered	16 (12-20)		15 (11-19)		.01	
	Sum of characters entered	253 (163-368)		208 (143-288)		<.001	
	Input time (minutes)	9.7 (6.4-15.5)		10.0 (6.6-16.2)		.50	

**Table 4:** Time required for nurses to enter patient data in the nursing system stratified by the patients' activities of daily living.

Characteristic		Use group	No-use group	*P* value
**Independent ADL^a^, median (IQR)**				
	Single-choice items registered	56 (50-63)	56 (51-64)	.33
	Multiple-choice items registered	16 (12-20)	15 (11-19)	.01
	Sum of characters entered	243 (160-353)	201 (138-272)	<.001
	Input time (minutes)	9.5 (6.4-15.0)	10.0 (6.5-16.5)	.20
**Dependent ADL, median (IQR)**				
	Single-choice items registered	54 (48-61)	54 (48-62)	.53
	Multiple-choice items registered	14 (10-19)	15 (10-19)	.89
	Sum of characters entered	232 (135-337)	234 (131-361)	.82
	Input time (minutes)	9.1 (5.8-13.0)	10.4 (6.6-18.0)	.006

^a^ADL: activities of daily living.

## Discussion

### Principal Results

This study developed an EMR-connected questionnaire system in which patients could answer electronic questionnaires inside and outside the HIS network, and the questionnaire response data were transmitted securely to the EMR servers. It also developed an intelligent quotation of the response data in the nursing system. Furthermore, most importantly, it demonstrated that these systems could reduce documentation time for nurses.

Nursing records include various patient assessments, vital signs, and nursing care records. This study focused on patient assessments at the time of admission. Overall, the study showed that quoting responses to electronic questionnaires reduced input time (Table 2). However, the content and volume of patient assessments may vary depending on diseases and patient ADL. Therefore, stratified analyses were performed. In the analyses of the internal medicine ward and patients with dependent ADL, there was no difference in the amount of information in the patient profiles generated by the 2 groups, that is, the number of items filled and the sum of characters entered in the text. The input time was 1.3-2 minutes shorter in the use group. Thus, if the volume of patient profiles created is the same, a quotation from the electronic questionnaire response in the patient profile would save 13%-18% (1.3-2 minutes) of the time. In terms of nurses' overall recording time, a reduction of 1 or 2 minutes may not be considered a significant impact. However, when this system is expanded to other nursing assessments, time savings can be expected. In the analyses of surgical wards and patients with independent ADL, there was no difference in input time, but the number of characters entered was significantly higher in the use group. This means that the quoting from the electronic questionnaire response resulted in a more complete description of the patient profile, despite the same amount of time. It has been reported that the time spent using electronic medical records is positively correlated with health care provider burnout [20-22]. Consequently, reducing the time spent documenting is meaningful. In addition, insufficient time for documentation is reported to be an independent predictor of burnout among nurses [23]. In light of this, it is significant for patients and nurses that more comprehensive descriptions were completed in the same amount of time, as observed in the surgical wards and in the patients with independent ADL in this study.

In terms of the nurses’ workflow, there may be differences between the use and no-use groups. In the no-use group, that is, the conventional method using paper questionnaires, the nurse typically receives the patient's completed questionnaire, additionally interviews the patient and adds what they heard to the questionnaire. Finally, the patient profile was launched on the EMR terminal, and all information was transcribed simultaneously. Conversely, in the use group, which used the EMR-connected questionnaire, nurses typically reviewed the patient profile that had already been partially filled out by quotation at the bedside while simultaneously conducting additional interviews with the patient and entering the spot. The input time measured in this study was the total time the patient profile was run on the EMR terminal. Additionally, the time spent interviewing patients while running the patient profile was included. Consequently, the difference in input time between the 2 groups might have been underestimated, although the workflow may not have been constant for individual nurses in either group.

Until now, there is a dearth of information regarding whether the integration of PROs and EHRs improves the efficacy of hospital operations. A randomized trial reported that collecting PROs in clinical studies using the EHR portal saves researchers time compared to collecting them by telephone [24]; however, it was a small-scale study and not conducted in the setting of general clinical practice. To the best of our knowledge, this is the first study to demonstrate, in a daily clinical setting and on a large scale, that importing PROs into an HIS network and quoting the data in a nursing system could reduce documentation time.

Among the sections comprising the patient profile, quotations from the EMR-connected questionnaire were frequently set up for those corresponding to the nutrition and metabolism, elimination, cognition or perception, and physical activity or exercise of Gordon's functional health pattern. These sections included assessments of the patient's activities, mobility in daily living, and sensory perception, such as vision or hearing. These matters were easy for the patient to answer in the electronic questionnaire, as they could answer whether they could do it. By contrast, quotations were not included in sections related to self-perception or self-concept. These sections focused on patients’ understanding of their feeling and attitudes toward the self. As patients do not usually have the opportunity to think about these matters, it was considered appropriate to assess them through direct conversations between the patient and nurse rather than through an electronic questionnaire. In addition, no quotes from the electronic interviews were set up in the form of roles or relationships. These sections focus on the details of the patient's family and the patient's involvement with them. These matters were considered difficult to ask in an electronic questionnaire because private information could often be included in the answers.

The series of systems in this study was realized using 2 chief technologies. The first is intelligent quotations. The PRO itself, which is not subject to interpretation by others, is important. However, transcribing PROs verbatim, whether in nursing records or progress notes, does not constitute medical records. Medical professionals must understand, interpret, classify, and supplement a patient's report from a professional perspective before classifying it as a medical record. Intelligent quotations make it possible to use data between documents of different natures, ranging from patient-oriented questionnaires to nursing records. Intelligent quotations will also play an important role in incorporating real-world data and patient-generated health data into medical and nursing records. This is because the necessary clinical indicators in the EMR often differ from the raw data provided by the patient, as in this study, in which the amount of alcohol consumed and alcohol units were calculated from the type and number of alcoholic beverages reported by the patient.

The other technology is the EMR-connected questionnaire system. It focused on ensuring the protection of patients’ data and security from access outside the hospital, which is a known concern in PRO and EHR integration [25]. The unique identifier and URL generated did not contain any personal information. The Outside-HIS Questionnaire server did not include any personally identifiable information. The patient questionnaire data were obtained from the HIS Questionnaire server in the HIS network and mapped to the hospital patient ID with the generated unique identifier. This allows the patient questionnaire data generated outside the network to be securely integrated into the EMR.

At present, the data captured in the HIS network are stored in XML format. In the future, interoperability can be ensured by adopting standards, such as the Health Level Seven Fast Healthcare Interoperability Resources.

This EMR-connected questionnaire system not only improves nurses' workflow and enhances nursing records but can also be used for nursing research in the future. Data can be efficiently collected on outpatients before and after nursing intervention and analyzed with EMR data.

### Limitations

The study has several limitations. It was a retrospective single-center study. The patients were not randomly assigned to use an electronic questionnaire or a traditional paper questionnaire. This may limit the generalizability of the findings. Nevertheless, the system allows patients to securely use the electronic questionnaire from both inside and outside the HIS network. The findings of this study can contribute to reducing nurses' recording time.

### Conclusions

The study developed and implemented a system in which self-reported patient data were captured in EMR and quoted in the nursing system. This study highlights that this system contributed to reducing nurses' recording time and enhanced the content of records. The system is expected to improve nurses' work efficiency and aid in clinical research without increasing patients’ burden.

## Appendix

Multimedia Appendix 1 Supplement tables for correspondence between Gordon's functional health pattern and the patient profile in the nursing system, and input tme analyses without excluding outliers.

## Abbreviations

ADL: activities of daily living
EHR: electronic health record
EMR: electronic medical record
HIS: hospital information system
ISO: International Organization for Standardization
PRO: patient-reported outcome
