# Supplementary material for: Impact of an Electronic Medical Record–Connected Questionnaire on Efficient Nursing Documentation: Usability and Efficacy Study
Source: JMIR Nurs. 2023 Sep 25;6:e51303. doi: 10.2196/51303 (PMC10562973; doi:10.2196/51303)
Supplement: Multimedia Appendix 1 [file nursing_v6i1e51303_app1.docx]

**Supplementary Paper for**

**Impact of Electronic Medical Record-Connected Questionnaire on Efficient Nursing Documentation: Usability and Efficacy Study**

Kana Kodama^1^, MMSc; Shozo Konishi^1^, MD, PhD; Shirou Manabe^1,2 *^ PhD; Katsuki Okada^1^, MD, PhD; Junji Yamaguchi^3^; Shoya Wada^1,2^, MD; Kento Sugimoto^1^, PhD; Sakiko Itoh^4^, PhD; Daiyo Takahashi^3^, MMSc; Ryo Kawasaki^5^, MD, MPH, PhD; Yasushi Matsumura^1,6^, MD, PhD; Toshihiro Takeda^1^, MD, PhD

1 Department of Medical Informatics, Osaka University Graduate School of Medicine, Osaka, Japan

2 Department of Transformative System for Medical Information, Osaka University Graduate School of Medicine, Osaka, Japan

3 MKS Inc., Osaka, Japan

4 Department of Home Health and Palliative Care Nursing, Graduate School of Health Care Sciences, Tokyo Medical and Dental University, Tokyo, Japan

5 Division of Public Health, Department of Social Medicine, Osaka University Graduate School of Medicine, Osaka, Japan

6 National Hospital Organization Osaka National Hospital, Osaka, Japan

* Deceased

*** Corresponding Author**

Shozo Konishi, MD, PhD

Division of Medical Informatics, Osaka University Graduate School of Medicine, Suita, Osaka, Japan

TEL: 81-6-6879-5900, Email: konishi.shozo.med@osaka-u.ac.jp

**Supplementary Paper**

**Methods**

**Intelligent Quotation of Questionnaire Response Data in Patient Profile**

The following table shows the correspondence between Gordon’s Functional Health Pattern and the patient profile in the nursing system and whether data are quoted from the patient questionnaire.

**Table S1. Correspondence table of Gordon's functional health pattern and the patient profile with and without quotes from the electronic questionnaire.**

| **Gordon’s Functional Health Pattern** | **Patient Profile** | **Quote from Electronic questionnaire** |
| --- | --- | --- |
| (1) Health perception/Health management | Medical history |  |
|  | History of patient illnesses |  |
|  | Nursing progress during hospitalization |  |
|  | Understanding medical care |  |
|  | History of self-medication errors |  |
|  | Smoking habit | ● |
|  | Drinking habit | ● |
|  | Fall Risk Assessment |  |
|  | Explanations from doctors about hospitalization and treatment for the patient |  |
|  | Understanding explanations from doctors about hospitalization and treatment |  |
|  | Adherence |  |
|  | Requests for doctors and nurses |  |
|  | Health management |  |
|  | Post-discharge expectations |  |
|  | Medical care after discharge |  |
|  | Medicine management |  |
| (2) Nutritional/Metabolic | Appetite | ● |
|  | Dietary restrictions | ● |
|  | Weight change | ● |
|  | Tooth problems | ● |
|  | Dysphagia | ● |
|  | Bedsores |  |
|  | Medical Device Related Pressure Ulcer (MDRPU) |  |
|  | Skin moisture |  |
|  | Skin edema |  |
|  | Skin tear |  |
|  | Protruding bone(s) |  |
|  | Special notes related to meals and nutrition |  |
| (3) Elimination | Defecation | ● |
|  | Number of night urination | ● |
|  | Excretion | ● |
|  | Special notes related to elimination |  |
| (4) Physical activity/Exercise | Disability related to walking | ● |
|  | Disability related to sitting and rolling over on the bed |  |
|  | Ability of self-positioning |  |
|  | ADL/ Eating | ● |
|  | ADL/ Dressing | ● |
|  | ADL / Ambulating | ● |
|  | ADL/ Transferring |  |
|  | ADL/ Toileting | ● |
|  | ADL/ Bathing | ● |
|  | IADL: Management of money |  |
|  | IADL: Preparation for meals |  |
|  | IADL: Housework (cleaning, washing, etc.) |  |
|  | IADL: Management of money |  |
|  | Use of assistive devices and braces |  |
|  | Dysfunction |  |
|  | Joint contracture |  |
| (5) Sleep/Rest | Sleeping time at home |  |
|  | Bruxism and snoring |  |
|  | Help to sleep |  |
|  | Special notes (e.g., the use of special medical devices such as CPAP) |  |
| (6) Cognition/Perception | Perception |  |
|  | Cognition |  |
|  | Visual impairment | ● |
|  | Seeing Aids | ● |
|  | Hearing impairment | ● |
|  | Hearing Aids | ● |
|  | Special notes (e.g., the utilization of social resources related to cognitive and sensory function) |  |
| (7) Self-perception/Self-concept | Personality |  |
|  | Anxiety |  |
|  | Attitude |  |
|  | Facial expression |  |
|  | Voice and speech pattern |  |
|  | Lack of concentration |  |
|  | Something/someone that helps the patient |  |
|  | Special notes |  |
| (8) Role/Relationships | Explanations from doctors about hospitalization and treatment for patient's family |  |
|  | Information about family: Family living together |  |
|  | Information about family: Separated family |  |
|  | Key person |  |
|  | Main caregivers |  |
|  | Family support during hospitalization |  |
|  | Daily routine |  |
| (9) Sexuality/Reproduction | Sexuality/reproduction |  |
| (10) Coping/Stress tolerance | Coping/stress tolerance |  |
| (11) Values/Beliefs | Values/beliefs |  |

ADL: Activities of daily living

IADL: Instrumental activities of daily living

**Results**

**Contribution to Reduction of Input Time for Patient Profile**

The following tables show the comparison results for the use and no-use groups when outliers were not excluded. TS2 and TS3 in this supplementary paper correspond to Tables 4 and 5, respectively, in the main paper.

**Table S2.** Time required for nurses to enter patient data into the nursing system without eliminating Input Time outliers.

| **Characteristic** | **Use group**  N = 1,326 | **No-Use** **group**  N = 1,099 | ***P* value** |
| --- | --- | --- | --- |
| Patient age (year), median (IQR) | 58 (40-73) | 56 (33-73) | .16 |
| Patient sex (male) | 614 (46) | 501 (46) | .72 |
| Independent ADL^a^ | 913 (84) | 713 (74) | <.001 |
| Prolonged hospital stay | 683 (54) | 536 (52) | .46 |
| Cognitive impairment | 91 (6.9) | 84 (7.6) | .47 |
| Single-choice items registered, median (IQR) | 56 (50-63) | 56 (50-63) | .25 |
| Multiple-choice items registered, median (IQR) | 16 (12-20) | 15 (11-19) | .02 |
| Sum of characters entered, median (IQR) | 237 (153-352) | 208 (139-302) | <.001 |
| Input Time (minutes) , median (IQR) | 9.7 (6.3-15.8) | 10.2 (6.7-17.2) | .038 |
| Ward |  |  | <.001 |
| Internal medicine | 614 (46) | 271 (25) |  |
| Surgery | 712 (54) | 828 (75) |  |
| Years working in nursing, median (IQR) | 4.7 (2.4-12.5) | 4.6 (2.3, 10.7) | .21 |
| EMR^b^ use duration > 3 years, n (%) | 862 (65) | 703 (64) | .59 |

^a^ADL: activities of daily living.

^b^EMR: electronic medical record.

**Table S3.** The time required for nurses to enter patient data in the nursing system was stratified by patient wards without excluding outliers of the Input Time.

| **Characteristic** | **Use group** | **No-Use group** | ***P* value** |
| --- | --- | --- | --- |
| Internal Medicine, median (IQR) |  |  |  |
| Single-choice items registered | 55 (49-63) | 54 (49-63) | .79 |
| Multiple-choice items registered | 15 (12-20) | 15 (11-18) | .38 |
| Sum of characters entered | 225 (144-336) | 212 (121-332) | .19 |
| Input Time (minutes) | 9.3 (6.1-15.5) | 11.0 (7.3-19.5) | .003 |
|  |  |  |  |
| Surgery, median (IQR) |  |  |  |
| Single-choice items registered | 56 (50-63) | 57 (51-64) | .47 |
| Multiple-choice items registered | 16 (12-20) | 15 (11-19) | .01 |
| Sum of characters entered | 250 (163-366) | 208 (143-294) | <.001 |
| Input Time (minutes) | 10.0 (6.4-16.1) | 10.1 (6.6-16.7) | .73 |

**Table S4.** The time required for nurses to enter patient data in the nursing system was stratified by patient activities of daily living without excluding outliers of the Input Time.

| **Characteristic** | **Use group** | **No-Use group** | ***P* value** |
| --- | --- | --- | --- |
| Independent ADL^a^, median (IQR) |  |  |  |
| Single-choice items registered | 56 (50-63) | 57 (51-64) | .32 |
| Multiple-choice items registered | 16 (12-20) | 15 (11-19) | .01 |
| Sum of characters entered | 243 (161-352) | 201 (137-273) | <.001 |
| Input Time (minutes) | 9.7 (6.4-15.8) | 10.0 (6.6-16.8) | .51 |
|  |  |  |  |
| Dependent ADL, median (IQR) |  |  |  |
| Single-choice items registered | 54 (47-61) | 54 (48-62) | .64 |
| Multiple-choice items registered | 15 (11-20) | 15 (10-18) | .75 |
| Sum of characters entered | 236 (141-357) | 235 (136-363) | .89 |
| Input Time (minutes) | 9.4 (5.8-15.4) | 11.0 (6.6-18.6) | .03 |

^a^ADL: activities of daily living.
